# Syringin: a naturally occurring compound with medicinal properties

**DOI:** 10.3389/fphar.2024.1435524

**Published:** 2024-07-22

**Authors:** Qingyuan Qian, Jinchao Pan, Jun Yang, Renjie Wang, Kai Luo, Zhenhui Wu, Shuhe Ma, Yuguang Wang, Maoxing Li, Yue Gao

**Affiliations:** ^1^ College of Pharmacy, Lanzhou University, Lanzhou, China; ^2^ Institute of Radiation Medicine Sciences, Beijing, China; ^3^ Faculty of Environment and Life, Beijing University of Technology, Beijing, China; ^4^ College of Pharmacy, Gansu University of Chinese Medicine, Lanzhou, China

**Keywords:** syringin, extraction and separation, pharmacological activities, preparations and applications, pharmacokinetics

## Abstract

Syringin, a phenylpropanoid glycoside, is widely distributed in various plants, such as *Acanthopanax senticosus* (Rupr. et Maxim.) Harms, *Syringa reticulata* (BL) Hara var. mandshurica (Maxim.) Hara, and *Ilex rotunda* Thumb. It serves as the main ingredient in numerous listed medicines, health products, and foods with immunomodulatory, anti-tumor, antihyperglycemic, and antihyperlipidemic effects. This review aims to systematically summarize syringin, including its physicochemical properties, plant sources, extraction and separation methods, total synthesis approaches, pharmacological activities, drug safety profiles, and preparations and applications. It will also cover the pharmacokinetics of syringin, followed by suggestions for future application prospects. The information on syringin was obtained from internationally recognized scientific databases through the Internet (PubMed, CNKI, Google Scholar, Baidu Scholar, Web of Science, Medline Plus, ACS Elsevier, and Flora of China) and libraries. Syringin, extraction and separation, pharmacological activities, preparations and applications, and pharmacokinetics were chosen as the keywords. According to statistics, syringin can be found in 23 families more than 60 genera, and over 100 species of plants. As a key component in many Chinese herbal medicines, syringin holds significant research value due to its unique sinapyl alcohol structure. Its diverse pharmacological effects include immunomodulatory activity, tumor suppression, hypoglycemic action, and hypolipidemic effects. Additionally, it has been shown to provide neuroprotection, liver protection, radiation protection, cardioprotection, and bone protection. Related preparations such as Aidi injection, compound cantharidin capsule, and Tanreqing injection have been widely used in clinical settings. Other studies on syringin such as extraction and isolation, total synthesis, safety profile assessment, and pharmacokinetics have also made progress. It is crucial for medical research to deeply explore its mechanism of action, especially regarding immunity and tumor therapy. Meanwhile, more robust support is needed to improve the utilization of plant resources and to develop extraction means adapted to the needs of industrial biochemistry to further promote economic development while protecting people’s health.

## Graphical Abstract

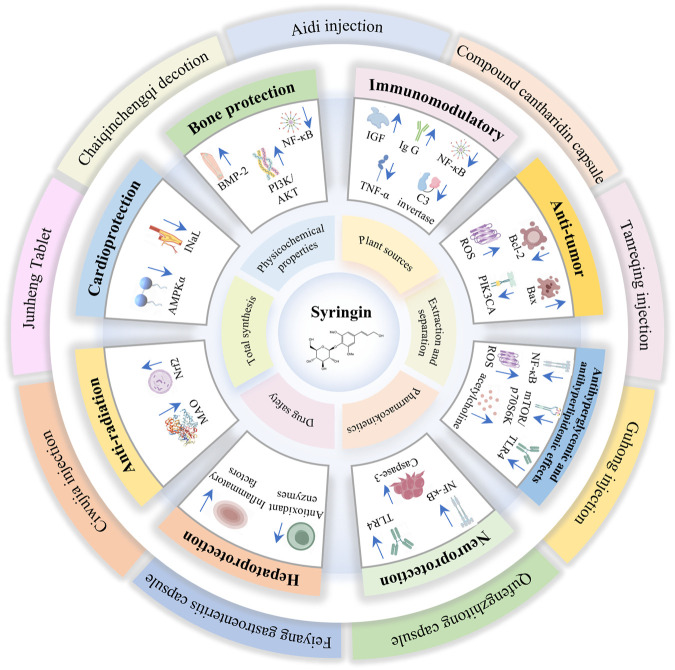

## 1 Introduction

Syringin (E−4-3-Hydroxy-1-propenyl-2, 6-dimethoxyphenyl-*β*-D-glucopyranoside; also known as Eleutheroside B) is a phenylpropanoid glycoside extracted from the roots of *A. senticosus* (Rupr. et Maxim.) Harms, a medicinal plant species traditionally used as folk medicine in Russia, China, Korea, and Japan (Davydov and Krikorian, 2000). Previous research has demonstrated that syringin possesses broad pharmacological effects, including immunomodulatory properties (Singh et al., 2023), anti-tumor activity (Wang et al., 2008), antihyperglycemic and antihyperlipidemic effects (Niu et al., 2007), neuroprotection (Tan et al., 2021), and hepatoprotection (Jiang et al., 2019) et al. Additionally, it meets all the criteria according to *Lipinski’s rule of five*. As all the values were within the acceptable range, it is evident that syringin can be considered a potential drug-like molecule (Ahmed et al., 2021). Furthermore, it serves as the main ingredient in various marketed drugs, health products, and foods such as Aidi injection (Yang et al., 2022), Compound cantharidin capsule (Sun et al., 2017), and Tanreqing injection (Wang L. et al., 2021).

Despite its incredibly high and comprehensive utilization values, syringin has not yet been fully explored. Lack of clarity regarding its action mechanism impedes further development and application of syringin-related formulations. It is essential to explore plant resources for syringin, enhance its resource utilization, and elucidate its pharmacological mechanism to promote the development and clinical application of products containing syringin. Thus, there is a need for comprehensive analysis to develop its potential further.

This article systematically reviews the physicochemical properties, plant sources, extraction and separation, total synthesis, pharmacological activities, drug safety, preparations and applications, and pharmacokinetics of syringin. The information on syringin was obtained from internationally recognized scientific databases through the Internet (PubMed, CNKI, Google Scholar, Baidu Scholar, Web of Science, Medline Plus, ACS Elsevier, and Flora of China) and libraries. Syringin, extraction and separation, pharmacological activities, preparations and applications, and pharmacokinetics were chosen as the keywords. The purpose is to provide valuable references for further expanding the research and application of this component.

## 2 Physicochemical properties

**FIGURE 1:**
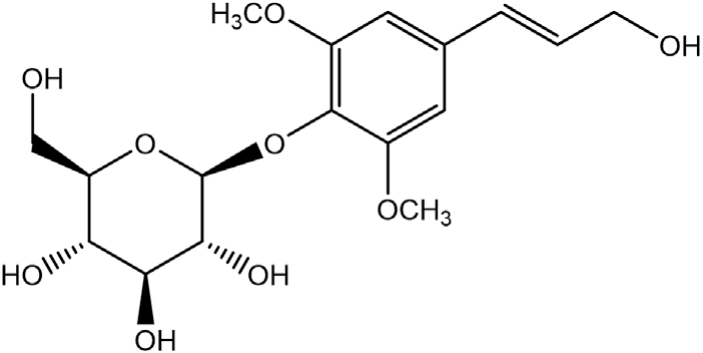
Structural formula of syringin.

Syringin is a phenylpropanoid glycoside with a molecular formula of C_17_H_24_O_9_, a relative molecular weight of 372.37, a density of 1.415 g cm^-3^, and a melting point of 192°C. Its chemical structural formula is shown in Figure 1. At room temperature, syringin exists as colorless needle crystals and is soluble in hot water, ethanol, and methanol. It is slightly soluble in cold water and acetone but insoluble in benzene, chloroform, and ether. When colored with 10% ethanol sulfate upon thin-layer inspection, syringin appears as purple spots (Mahadeva et al., 2015). The ultraviolet (UV) spectrum (MeOH) shows λ max (log ϵ) at 221 (4.50) nm and 266 (4.19) nm (Min et al., 2004). The infrared ray (IR) spectrum data for syringin can be found in Table 1. However, it should be noted that the data is outdated and may require updating by relevant researchers. Furthermore, Table 2 presents the mass spectrometry data for syringin for reference purposes.

**TABLE 1:** IR spectrum data of syringin.

NO.	Frequency (cm^-1^)	Vibration type, functional group
1	3,570	ms, 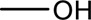
2	3,385.3	vs., 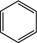 , 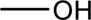
3	3,300–3,000	vw, 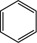 , 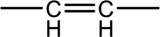
4	2921.8	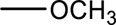 , 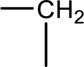 , 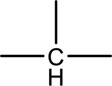
5	1584.9, 1508.5	ms, 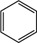
6	1464.7	ms, 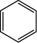 , 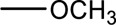 , 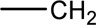
7	1418.3	ms, 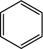 , 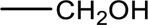 , 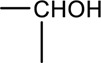
8	1241.7	ms, 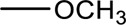 , 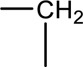 , 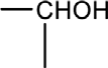
9	1133.2	ms, 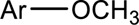 , 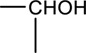 , 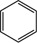
10	964.9	ms, 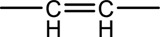
11	844.2	m, 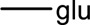 Ⅱ 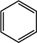
12	784.4	m, 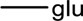 Ⅲ 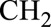
13	630.3	m, 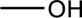 , 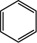 , 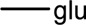

**TABLE 2:** ^13^C and ^1^H NMR data of syringin.

Position	Li et al. (2018)		Noshita et al. (2021)	
	δ_H_ (*J* in Hz)	δ_C_	δ_H_ (*J* in Hz)	δ_C_
1	6.78, s	135.9	6.74, s	135.8
2		154.4		154.4
3		105.5		105.5
4		135.3		135.3
5	6.78, s	105.5	6.74, s	105.5
6		154.4		154.4
7	6.58, dt (15.9,1.6)	131.3	6.54, d (15.9)	131.3
8	6.35, dt (15.9,5.6)	130.0	6.32, dt (15.8,5.6)	130.0
9	4.25, dd (5.6,1.6)	63.6	4.21, dd (5.5,1.1)	63.6
-OCH3	3.89, s	57.0	3.85, s	57.0
1′	4.90, d (7.5)	105.4	4.85, d (7.6)	105.4
2′	3.41–3.54 (overlap)	75.7	3.51–3.37, m	75.7
3′	3.41–3.54 (overlap)	78.4	3.51–3.37, m	78.4
4′	3.41–3.54 (overlap)	71.4	3.51–3.37, m	71.3
5′	3.24, m	77.8	3.24–3.15, m	77.8
6′a	3.81, dd (11.9, 2.4)	62.6	3.77, dd (12.0, 2.3)	62.6
6′b	3.69, dd (11.9, 5.2)		3.65, dd (12.0, 5.1)	

## 3 Plant sources

**FIGURE 2:**
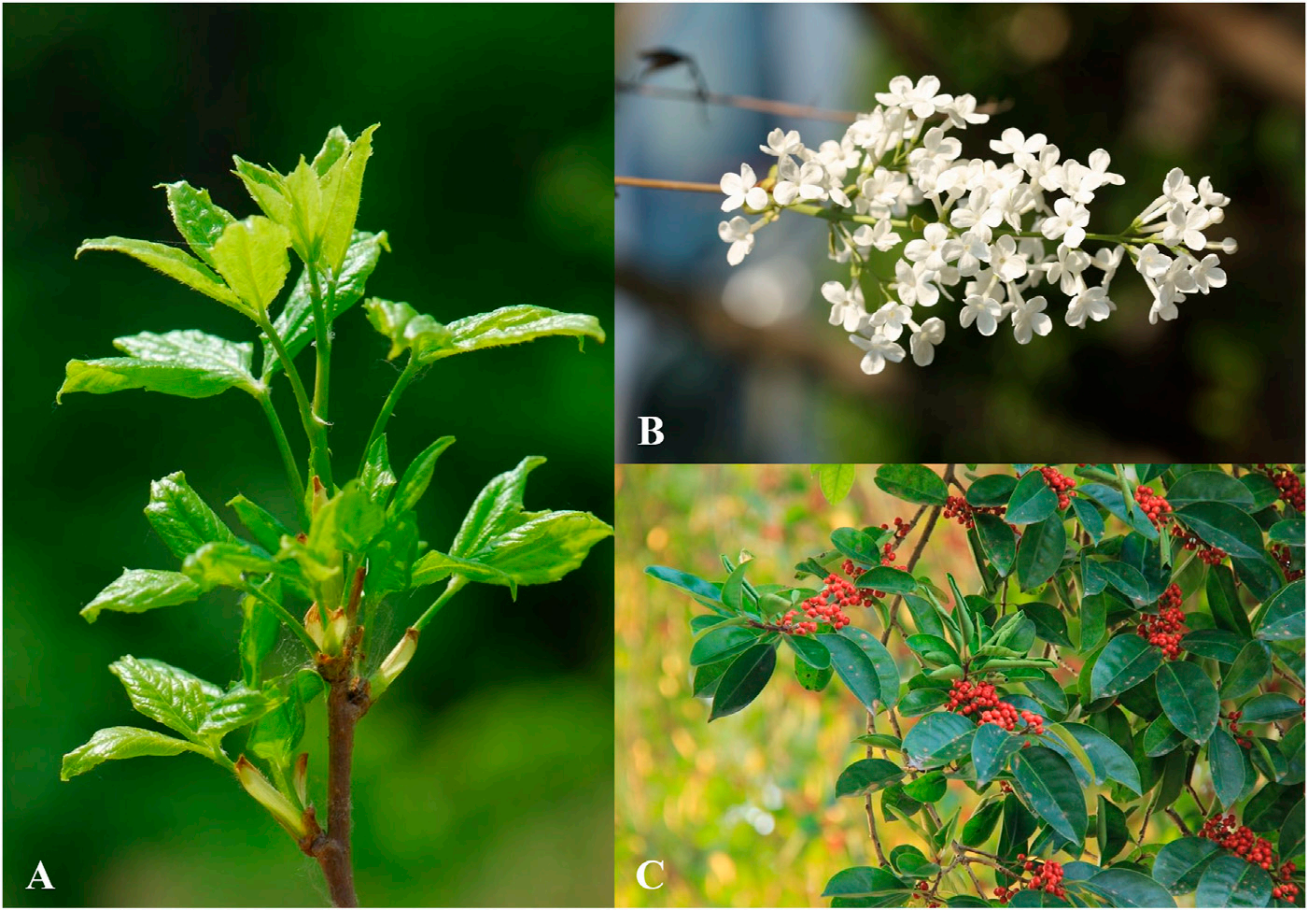
**(A)** *A. senticosus* (Rupr. et Maxim.) Harms; **(B)** *S. reticulata* (BL) Hara var. mandshurica (Maxim.) Hara; **(C)** *I. rotunda* Thumb. (The copyright of these pictures was obtained from https://www.iplant.cn/frps/vol).

Syringin is a bioactive compound isolated from numerous pharmacologically essential plant species. According to statistics (Wang F. et al., 2021), syringin is found in 23 families, over 60 genera, and over 100 plant species, some of which are listed in Table 3. The data indicates that the content of syringin is relatively high in the phloem of *S. reticulata* (BL) Hara var. mandshurica (Maxim.) Hara and *I. rotunda* Thumb (Figure 2).

**TABLE 3:** Some plant resources with syringin.

NO.	Family	Genera	Species	Part and content
1	Oleaceae Hoffmanns. and Link	S. Linn	*S. reticulata* (BL) Hara var. mandshurica (Maxim.) Hara	Stem bark (3.59%), stem xylem (0.10%), root (0.42%) (Zhang et al., 2010a)
2			*S. dilatata*	Stem bark (1.53%), stem xylem (0.19%) (Mun and Mun, 2015)
3			*S. Pubescens* Turcz	Stem bark (1.15%) (Xu, 2021)
4	Aquifoliaceae	I. L	*I. rotunda* Thumb	Stem bark (3.53%), leaves (0.42%), fruits (0.40%) (Wang et al., 2016)
5	Crassulaceae J. St.-Hil	Rhodiola L	*Rhodiola crenulate* (Hook.f.et Thoms.) H. Ohba	Root and rhizome (3.02%) (Yang, 2016)
6	Cornaceae Bercht. and J. Presl	Toricellia DC.	*Toricellia Angulata* Oliv. var. intermedie (Harms) Hu	Root phloem (0.56%) (Han et al., 2017)
7	Araliaceae Juss	Eleutherococcus Maxim	*A. senticosus* (Rupr.et Maxim.) Harms	Stem (0.32%) (Zhang et al., 2010b)
8		Aralia L	*Eleutherococcus sessiliflorus* (Rupr. and Maxim.) S.Y. Hu	Stem (0.08%) (Zhang et al., 2010b)
9		Lysionotus D. Don	*Aralia decaisneana* Hance	Root phloem (0.17%) (Zhou et al., 2013)
10			*Acanthopanar gracilistylus* W. W. Smith	Root phloem (0.09%) (Wang et al., 2011)
11	Thymelaeaceae Juss	Daphne Linn	*Daphne tangutica* Maxim	Whole-plant (0.25%) (Wang and Li, 2018)
12		Edgeworthia Meisn	*Edgworthia chrysantha*	Whole-plant (0.18%) (Tang and Tang, 2014)
13	Buxaceae Dumort	Buxus L	*Buxus microphylla Sieb*.et Zucc. var. sinica Rehd.et Wils	Thick stem (0.03%), twig (0.05%) (Yang et al., 2017)
14	Asteraceae Bercht. and J. Presl	Saussurea DC.	*Saussurea involucrate* (Kar.et Kir.) Sch.Bip	Aboveground (0.06%) (Zhai et al., 2008)
15	Eucommiaceae	Eucommia Oliv	*Eucommia ulmoides* Oliv	leaves (0.05%) (Zhang et al., 2019)
16	Magnoliaceae Juss	Magnolia L	*Magnolia officinalis* Rehd.et Wils	leaves (0.04%) (Wu et al., 2018)
17	Campanulaceae Juss	Codonopsis Wall	*Codonopsis tangshen* Oliv	Root (0.04%) (Hu, 2017)
18	Loranthaceae Juss	Viscum L	*Vhcum coloratum* (Komar.) Nakai	Leafy stem branch (0.02%) (Gao et al., 2018)

Among these plants, *A. senticosus* (Rupr. et Maxim.) Harms, also known as Siberian ginseng, is a medicinal and edible plant with multiple biological activities. This popular medicinal plant is widely used in China and Russia and is one of the top ten herbal dietary supplements in the United States (Davydov and Krikorian, 2000). Its functions and indications have been recorded in the *Compendium of Materia Medica* and the *Records of Famous Doctors* throughout various dynasties. *Acanthopanax senticosus* (Rupr. et Maxim.) Harms is warm, spicy, slightly bitter, non-toxic, and belongs to the spleen and kidney meridians. It is commonly used in traditional Chinese medicine for symptoms such as heart and body deficiency, spleen and kidney yang deficiency, insomnia, and excessive dreaming (Chen et al., 2024). Due to its significant health benefits, *A. senticosus* (Rupr. et Maxim.) Harms have been processed into herbal tea and capsules for daily use (Hu et al., 2024). According to the United States Pharmacopoeia (USP) and the European Pharmacopoeia standards (Guo et al., 2014), one of the quality requirements is that the content of syringin should be more than 0.8%.

## 4 Extraction and separation

The extraction, separation, and purification processes of syringin are complex and costly, resulting in low yields that limit its application in medicine and clinics.

He (2022) utilized ethanol reflux extraction to extract syringin from *A. senticosus* (Rupr. et Maxim.) Harms. The optimal extraction conditions included a 75% ethanol concentration, an extraction time of 1.5 h, and petroleum ether, chloroform, and ethyl acetate for extraction, followed by silica gel column chromatography for purification and isolation. The purity of the extracted syringin was determined to be 95.19%, providing a solid research foundation for potential industrial applications.

Duan et al. (2014) developed a method for preparing syringin from the *I. rotunda* Thumb. The process involves the following steps: 1) Crushing *I. rotunda* Thumb to 20–50 mesh, extracting it with water, and concentrating it by centrifugation to obtain the upper column solution; 2) Passing the upper column solution through a resin column and eluting it with water and 10%–40% ethanol (v/v). The eluates were collected in sections; 3) Concentrating the eluted portion of 10%–40% ethanol (v/v) by membrane separation and obtaining the product by spray drying. The method is simple, feasible, cost-effective, and suitable for industrial production.

Yan et al. (2004) successfully isolated syringin from the n-butanol extract of the stems and barks of *Edgeworthia chrysantha* Lindl for the first time. The extraction process involved a two-phase solvent system consisting of ethyl acetate-ethanol-water at a volume ratio of 15:1:15 (v/v/v), which was optimized for this study. Preparative high-speed counter-current chromatography isolated 28 mg of syringin with over 96% purity from 110 mg of the partially purified extract, as confirmed by high-performance liquid chromatography (HPLC) analysis.

Ethanol reflux extraction is commonly utilized in laboratory research because it effectively extracts various chemical components. However, the properties of different compounds vary, leading to a generally low extraction rate as the optimal conditions for each compound cannot be precisely defined. The primary goal of researching the extraction of Chinese medicinal materials or monomer compounds is to facilitate their clinical use. The extraction and separation technology for the syringin monomer still needs further development, emphasizing the necessity to enhance extraction methods to improve its pharmacological effects.

## 5 Total synthesis

Due to the significant pharmacological effects of syringin and its potential for clinical application, as well as the need for further development in extraction and isolation methods, the synthesis process holds crucial research value in medicinal chemistry.

**FIGURE 3:**
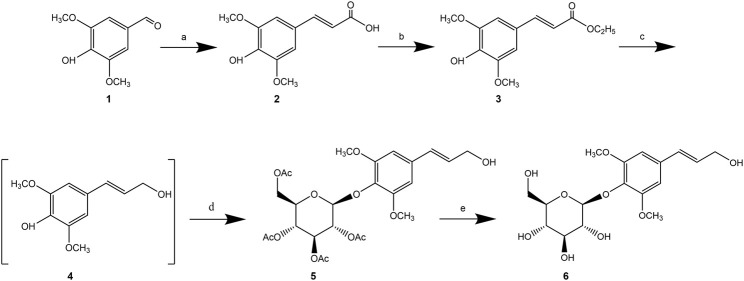
Synthetic route of syringin (Dong et al., 2021).

For instance, Dong et al. (Dong et al., 2021) described a 5-step synthesis of syringin using Doebner-Knoevenagel condensation of syringaldehyde with malonic acid as the key step to form the α, β-unsaturated carboxylic acid. The final yield of syringin was reported to be 54% (Figure 3).

**FIGURE 4:**
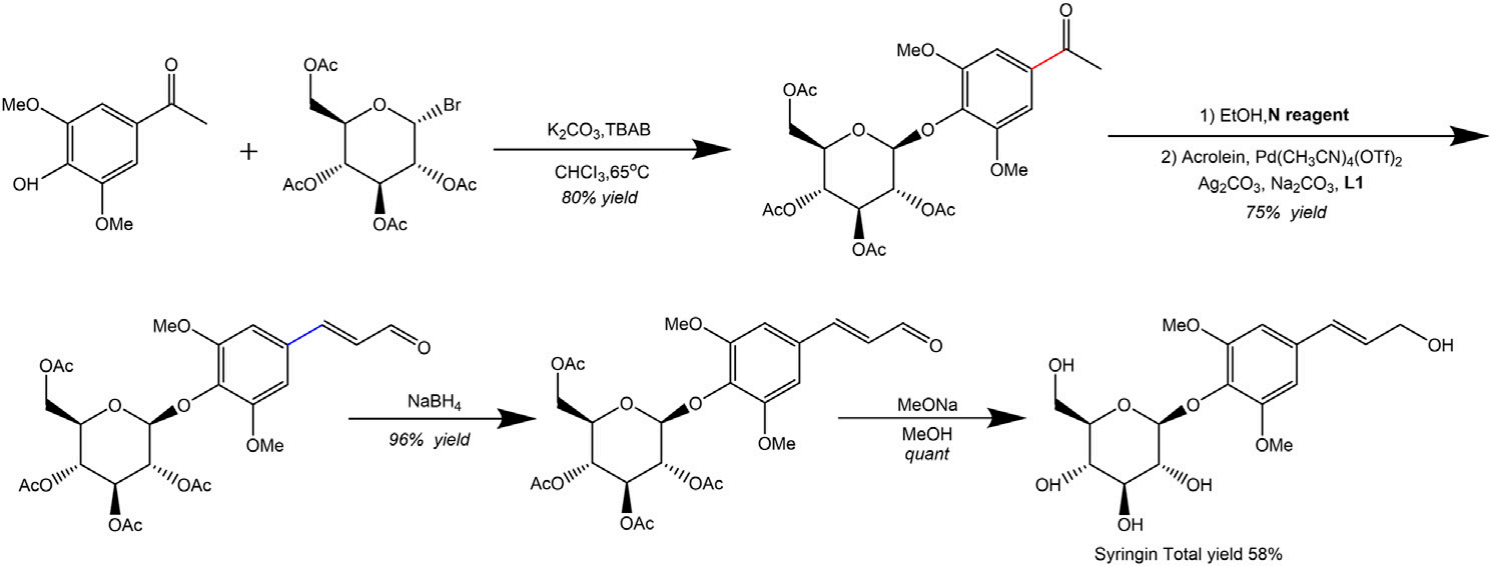
Synthetic route of syringin (Wang et al., 2023).

Additionally, Wang et al. (2023) synthesized natural product syringin from commercially available starting materials in five steps with an overall yield of 58%. The palladium-catalyzed C(O)-C bond activation of aryl ketone was identified as a crucial step in the total synthesis process. Furthermore, various syringin analogues bearing alkynyl, alkenyl, aryl, alkyl, siliconyl, and boronyl groups were also constructed via the aryl palladium intermediate (Figure 4).

## 6 Pharmacological activities

Syringin exhibits significant pharmacological effects, including immunomodulatory, anti-tumor, antihyperglycemic, and antihyperlipidemic effects. It also provides neuroprotection, hepatoprotection, anti-radiation, cardioprotection, and bone protection. These properties suggest the potential for syringin to be developed into drugs and health products (Table 4). Its mechanism of action is primarily associated with the elimination of oxidative free radicals, enhancement of antioxidant enzyme activity, inhibition of inflammatory signaling pathways activation, and suppression of inflammatory factor production (Shen et al., 2020; Dai et al., 2021; Tan et al., 2021).

**TABLE 4:** Pharmacological activities of syringin.

Pharmacological activities	Subjects	Syringin doses	Mechanism of action	Ref.
Immunomodulatory	Croton oil-, arachidonic acid- and fluorescein-isothiocynate -induced mouse ear oedema model; LPS-stimulated RAW264.7 cells	Syringin (0.7, 7, 70, and 700 mg/kg); (62.5, 125, 250, 500, and 1000 µM)	Inhibited TNF-α production and cytotoxic T cell proliferation	Cho et al. (2001)
	Female BALB/c mice	Syringin (25, 50, and 100 mg/kg)	Improved asthma symptoms in ovalbumin-induced mice by modulating NF-κB pathway activation	Dai et al. (2021)
	DSS-induced colitis model; LPS-induced rat small intestine crypt epithelial (IEC6) cells	Syringin (100 mg/kg); Syringin (12.5 and 25 μg/mL)	Inhibited NF-κB and activated the Nrf2 signaling pathway in colitis	Zhang et al. (2020)
	LPS and D-GalN-induced FHF in mice	Syringin (10, 30, and 100 mg/kg)	Inhibited NF-κB activation and reduced TNF-α production	Gong et al. (2014)
	IPEC-J2 cells	Syringin (0, 0.05, 0.10, and 0.20 mg/mL)	Decreased cellular membrane permeability and mRNA expression of proinflammatory cytokines, increased tight junction protein expression and anti-inflammatory cytokines	Che et al. (2019)
	Rat adjuvant-induced arthritis and anterior cruciate ligament transection-induced OA rats	Syringin (50, 100, and 200 mg/kg)	Selectively inhibited COX-2	Chen et al. (2021b)
Anti-tumor	HGC-27 cells	Syringin (90, 180, and 360 µM)	Induced apoptosis by upregulating Bax protein expression and downregulating Bcl-2 protein expression. Inhibited tumor cell proliferation by arresting the cell cycle in the G0/G1 phase	He (2022)
	S180 tumor-bearing mice	Syringin (200 and 400 mg/kg)	Inhibited the growth of solid tumors, and the tumor inhibition rates were 61.16% and 25.89%, respectively	Wang et al. (2010b)
	Human breast cancer (MDA-MB-231 and MCF-7) cells	Syringin (0, 20, 40, 80, 160, and 320 μg/mL)	Against BC via PI3K-AKT-PTGS2 and EGFR-reticular activating system- Recombinant C-Raf Proto Oncogene Serine - mitogen-activated protein- extracellular regulated protein kinases (RAS-RAF-MEK-ERK) pathways	Wang et al. (2022a)
	AuNP modified by bxpc3 pancreatic cancer (BxPC3) and human hepatocellular carcinoma (Huh7) cells	G. diversifolia methanolic extract	BxPC3 (IC50 = 12.5 μg/mL); Huh7 (IC50 = 7.2 ± 2.5 μg/mL)	Aventurado et al. (2020)
	Colon adenocarcinoma (Caco-2) cells	Syringin (10 µM)	Inhibited hCAIX and hXII.	Costa et al. (2020)
	Non-tumorigenic, tumorigenic and metastatic BC cell lines; mouse model of xenograft tumor	Syringin (1, 3, 10, 30, and 100 mΜ); (5 mg/kg)	Upregulated cyclin-dependent kinase inhibitor 1 A (p21), cleaved cysteinyl aspartate specific proteinase (caspase-3/caspases-9), and PARP but downregulated cyclin-dependent kinase 4 (CDK4) and X-linked inhibitor of apoptosis protein expression concomitant with the suppression of growth in breast carcinoma cells, also induced excessive ROS levels in BC cells	Lee et al. (2019)
Antihyperglycemic and antihyperlipidemic effects	Wistar rats	Syringin (100 g/kg)	Increased the ability of nerve endings to release acetylcholine, stimulated the M3 receptor in pancreatic cells, and increased insulin release, resulting in a hypoglycemic effect	Niu et al. (2007)
	High glucose-induced mouse podocytes	Syringin (2.5, 5, and 10 µM)	Inhibited activation of mTOR/70 kDa Ribosomal Protein S6 Kinase 2 (p70S6K) pathway	Yao et al. (2023)
	Type-2 diabetic SD rats Rat myocardial (H9C2) cells treated with high glucose	Syringin (60 mg/kg) Syringin (15 µM)	Downregulated the TLR4/MyD88/NF- κB pathway and upregulated the peroxisome proliferator-activated receptor γ coactivator-1α (PGC1)/SIRT3 mitochondrial protection pathway	Yao et al. (2021)
	Conscious rats under chemical sympathectomy using an intraperitoneal injection of guanethidine	Syringin (100 μg/kg)	The hypoglycemic effect was related to the sympathetic nerve	Niu et al. (2008a)
	Mice fed on a high-fat diet	Syringin (5 mg/kg)	Through the production of adiponectin, it suppressed chronic inflammation and promoted the activation of skeletal muscle AMPK, thus reducing lipotoxicity and endoplasmic reticulum stress	Kim et al. (2017)
	Mouse embryonic fibroblast (3T3-L1) cells	Syringin (20 µM)	Enhanced the phosphorylation of AMP-activated protein kinase and acetyl-coenzyme A carboxylase, thus inhibiting adipocyte differentiation and adipogenesis and promoting fat metabolism	Hossin et al. (2021)
	Human breast cancer (MDA-MB-231 and MCF-7) cells	Syringin (10 µM); (6.8 µM)	Activated adiponectin receptor	Sun et al. (2013)
Neuroprotection	Human neuroblastoma cell (SK-N-SH and SK-N-BE) cells treated with amyloid β fragment Aβ (25-35)	Syringin (0, 5, 10, and 20 µM)	Enhanced the expression of miR-124-3p and decreased the level of BID.	Zhang et al. (2021a)
	The middle cerebral artery embolism model was established in SD rats	Syringin (5, 10, and 20 mg/kg)	Reduced rat cerebral ischemia damage and improved nerve function damage through the FOXO3α/NFκB axis	Tan et al. (2021)
	Cerebral ischemia/reperfusion injury in rats	Syringin (50 mg/kg)	Attenuated inflammation and brain injury in rats with ischemia-reperfusion injury by inhibiting the TLR4 receptor	Liu et al. (2022)
	Patch clamp-treated HEK-293 cells	Syringin (6.25, 25, and 100 µM)	Suppressed VRAC channel	Xu et al. (2023)
	Cortical neurons of rats injured by Ab (25-35)	Syringin (1 and 10 µM)	Ab-induced axonal and dendritic atrophy of cortical neurons in rats	Bai et al. (2011)
	Rat neuroblastoma cells treated with Aβ(25-35)	Syringin (25 µM)	Reduced the activity and expression of Caspase-3, reduced PARP, and made DNA fragmented	Yang et al. (2010)
	Human neuroblastoma (SH-SY5Y) cells treated with 6-OHDA	Syringin (2 and 4 µM)	Regulated miR-34α/SIRT1/BECN-1 axis to induce autophagy and prevent 6-OHDA-induced apoptosis and α-synuclein accumulation	Fu et al. (2023)
Hepatoprotection	HBV mice	Syringin (10 mg/mL)	Reduced liver oxidative stress, enhanced immunity, and prevented HBV replication	Jiang et al. (2019)
	Acute liver injury induced by LPS/D-GalN in mice	Syringin (25 and 50 mg/kg)	Inhibited the activation of NF- κB p65 protein in the liver, inhibited the production of inflammatory factors IL-6, IL-1β, TNF- α, and promoted the production of antioxidant enzymes in the liver	Hu et al. (2023)
	Acute liver injury model established by human hepatocyte (L-02) cells	Syringin + costunolide (10 + 40 µM)	Decreased the production of alanine aminotransferase, aspartate aminotransferase, lactate dehydrogenase, MDA, and ROS, increased the activity of superoxide dismutase and catalase, decreased the expression of Caspase-3, 7, 9, NF- κB, TNF-α, Cyclin B, recombinant cyclin-dependent kinase 1 (CDK1), and protein kinase inhibitors kappa-B (PKI) κB. The anti-acute liver injury effect was induced by ras-related C3 botulinum toxin substrate 1 (Rac1)/Akt/NF- κB signal pathway	Mao et al. (2023)
Anti-radiation	Injury model of mice induced by low-dose radiation	Syringin (10 and 20 mg/kg)	Regulated the disorder of cytokines in the testis of mice, improved the level of oxidative stress in the testis, and restored the testosterone level in the testis of mice	Hu et al. (2023)
	Injury model of mice induced by low-dose radiation	Syringin (20 mg/kg)	Protected spleen and thymus damage	Mei (2023)
	A mouse model of brain injury induced by^60^Co-γ irradiation	Syringin (240 μg/kg)	Prevented damage to the learning and memory ability of irradiated mice, protected the neurons of irradiated mice, improved the antioxidant activity of irradiated mice, and changed the level of neurotransmitters in irradiated mice	Song et al. (2022)
Cardioprotection	Model of atrial fibrillation induced by sea anemone toxin II in rabbit atrial myocytes	Syringin (200 µM)	Inhibited cell electrophysiological activity	Zhang et al. (2021b)
	Aortic banding-induced cardiac hypertrophy in mice	Syringin (50 and 100 mg/kg)	Decreased the protein expression levels of recombinant autophagy-related protein 7 (ATG7), BECN1, prostacyclin (p62), and autophagy-related protein LC3 A/B (LC3A/B), and decreased the phosphorylation level of AMPKα	Li et al. (2017)
	Rat intestinal microorganism	Syringin (10 mg/mL)	Stimulated intestinal bacteria to produce short-chain fatty acids, thus further promoting the role of myocardial ischemia	Yu et al. (2022)
Bone protection	Fully differentiated human osteoblast (Hob) cells	Syringin (60, 80, and 100 µM)	Increased alkaline phosphatase activity, increased mineral sedimentation, and regulated autophagy and the BMP-2 signal pathway	Imtiyaz et al. (2020)
	Ovariectomized mice	Syringin (10, 20, and 40 mg/kg)	Prevented bone loss by TNF receptor-associated factor 6 (TRAF6)-mediated inhibition of NF-κB and stimulation of PI3K/AKT, subsequently increasing the recombinant osteoprotegerin/receptor activator of nuclear kappa-B (OPG/RANKL) ratio and inhibiting osteoclastogenesis, finally promoting bone formation	Liu et al. (2018)
	Osteoarthritis induced by transection of the anterior cruciate ligament in rats	Syringin (25, 50, and 100 mg/kg)	Decreased the levels of IL-6, TNF- α, and IL-1β in serum and joint lavage fluid; decreased the protein expression levels of matrix metalloproteinase-13 (MMP-13), Wnt1, and β-catenin	Qiao et al. (2022)

### 6.1 Immunomodulatory

The primary function of the immune system is to recognize and eliminate microorganisms, foreign cells, or macromolecules that invade the body, as well as to remove cells with altered surface antigens to protect the body from harm. When immune cells identify a foreign body and migrate to the damaged area, they initiate an inflammatory response to eliminate the foreign body and promote tissue repair. Developing immune-enhancing agents with fewer adverse effects and lower costs holds excellent promise. Syringin has been identified as an effective natural immunostimulant for ameliorating immune-related diseases (Singh et al., 2023).

Research has indicated that syringin can inhibit the classical activation pathway of complement C3 convertase and *in vitro* immune hemolysis of sheep red blood cells coated with guinea pig serum antibodies. It also demonstrates a dose-dependent effect. Additionally, syringin can significantly increase the content of immunoglobulin G in serum and activate macrophages, thereby exerting further immune regulatory effects (Kapil and Sharma, 1997). In a study by Dai et al. (2021), a mouse asthma model was established, revealing that syringin can inhibit airway inflammation and the onset of asthma, suggesting its potential anti-inflammatory effects. Furthermore, it can reduce levels of peroxidation products such as malondialdehyde (MDA) and myeloperoxidase, thus preventing additional damage to inflammatory tissues.

Zhang et al. (2020) discovered that syringin has the potential to exert anti-inflammatory effects by inhibiting the overproduction of interleukin-1β (IL-1β), interleukin-6 (IL-6), tumor necrosis factor-α (TNF-α), and cyclooxygenase-2 (COX-2) induced by dextran sulfate sodium (DSS) or lipopolysaccharide (LPS). Additionally, it was found to hinder the nuclear factor kappa-B (NF-κB) p65 pathway by reducing the phosphorylation of the NF-κB inhibitor alpha (IκBα) Ser 32 site and promoting the activation of antioxidant nuclear factor erythroid 2-related factor 2 (Nrf2) signaling pathway, thereby exerting protective effects against intestinal inflammation. Syringin also dose-dependently inhibited the proliferation of mouse leukemia cells of monocyte macrophage (RAW264.7), TNF-a production, and mouse T cell CTLL-2 stimulated by LPS, demonstrating an antiallergic effect. However, high concentrations of syringin did not inhibit either NO or CD4 T cell proliferation (Cho et al., 2001). Studies have shown that syringin may attenuate LPS/D-galactosamine (D-GalN)-induced fulminant liver failure (FHF) by inhibiting NF-κB activation and reducing TNF-α production (Gong et al., 2014). It was claimed that syringin increased intestinal barrier function, tight junction protein expression, and anti-inflammatory cytokines while decreasing pro-inflammatory cytokine synthesis in porcine intestinal epithelial (IPEC-J2) cells (Che et al., 2019). Furthermore, it has been suggested that c-Jun N-terminal kinases 3 may be a key target for the anti-inflammatory properties of syringin (Geng et al., 2022). Syringin is expected to be developed as a COX-2 selective inhibitor for treating rheumatoid arthritis (RA) and osteoarthritis (OA) (Chen Q. et al., 2021).

**FIGURE 5:**
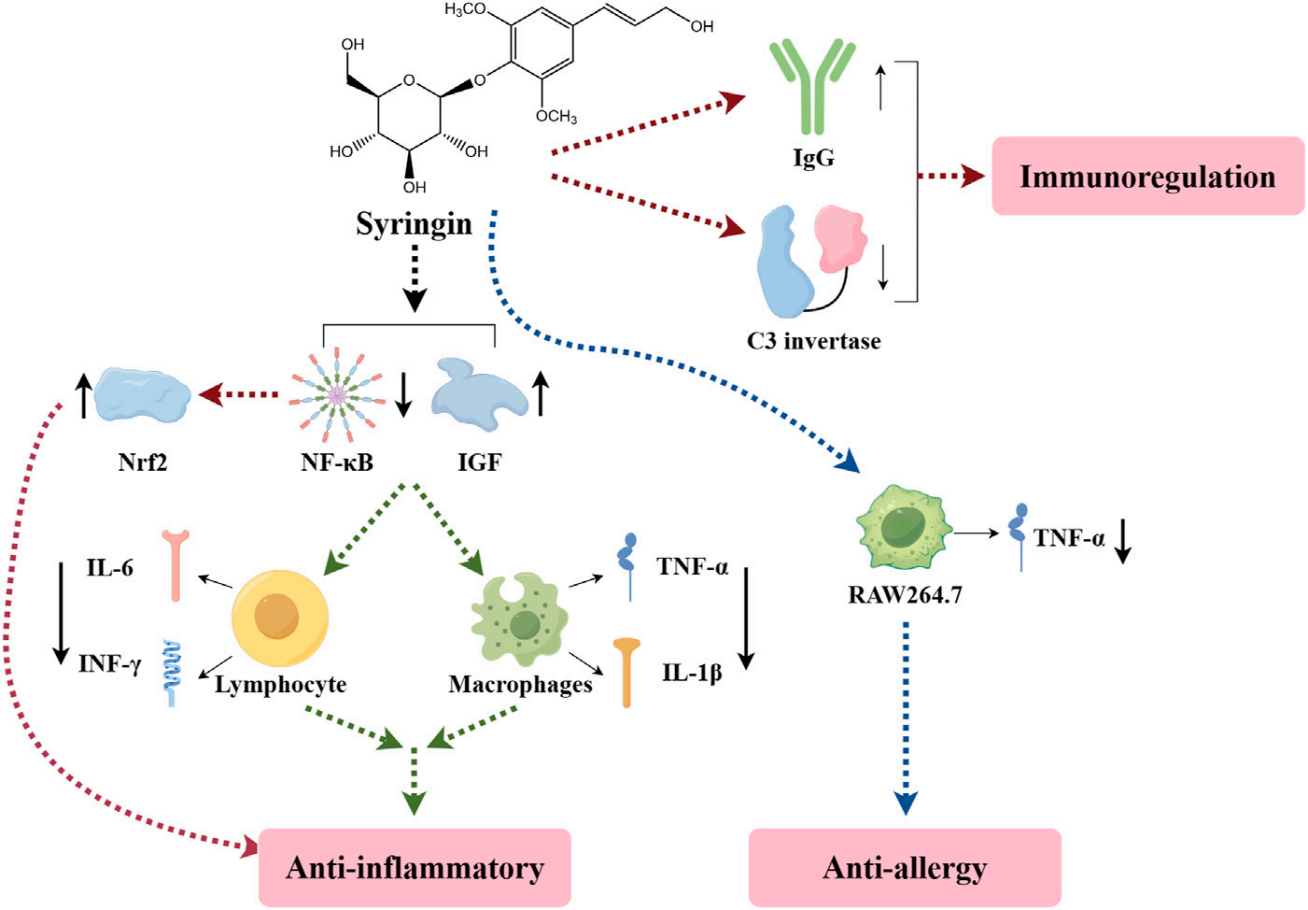
Mechanism of immunomodulatory effects of syringin.

These studies have demonstrated that syringin exhibits immunomodulatory effects primarily through the activation of cytokines, downregulation of the NF-κB signaling pathway, and upregulation of the Nrf2 and insulin-like growth factor signaling pathways (Figure 5). These findings suggest that syringin holds excellent potential for treating inflammation-related disorders and enhancing macrophage phagocytosis.

### 6.2 Anti-tumor

**FIGURE 6:**
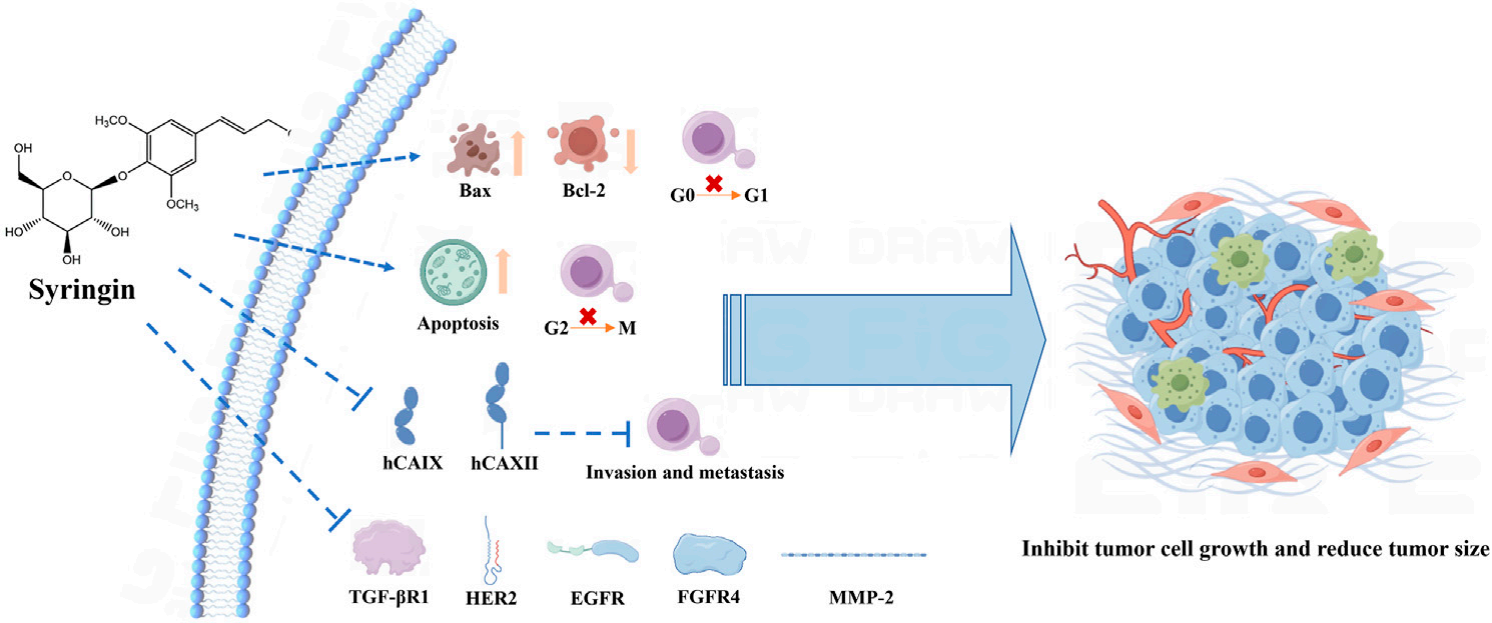
Mechanism of anti-tumor effects of syringin.

Currently, chemotherapeutic drugs used for treating tumors often lead to severe adverse reactions. This has led to a new trend in extracting effective anti-tumor natural ingredients from traditional Chinese medicine. Syringin, in particular, has shown promising anti-tumor effects (Figure 6). Further investigation into the related molecular mechanism can provide a solid theoretical foundation for the development of clinically targeted eugenol drugs (Wang F. et al., 2021).

Syringin exhibited significant inhibitory effects on human cervical cancer (HeLa) cells, human breast cancer (MCF-7) cells, human non-small cell lung cancer (A549) cells, and human prostate cancer (PC-3) cells, with an apparent quantity-efficacy relationship observed in all cases. Furthermore, experiments conducted on tumor-bearing mice demonstrated that syringin could effectively suppress the growth of solid tumors, with tumor inhibition rates of 61.16% and 25.89% for the high and low-dose groups, respectively (Wang Z. et al., 2010). Additionally, syringin was found to effectively inhibit the proliferation of human gastric cancer (HGC-27) cells through a mechanism involving significant cytotoxicity. It induces apoptosis by up-regulating Bcl-2-associated X protein (Bax) protein expression while down-regulating B-cell lymphoma-2 (Bcl-2) protein expression and inhibiting cell proliferation by blocking the cell cycle at the G0/G1 phase (He, 2022). Studies have also indicated that syringin exerts inhibitory effects on HeLa cells by inducing apoptosis, blocking the G2/M cell cycle, and inhibiting cell migration (Xia, 2016). Wang F. et al. (2022) identified mitogen-activated protein kinase 1 (MAP2K1), phosphatidylinositol-4,5-bisphosphate 3-kinase catalytic subunit alpha (PIK3CA), recombinant V-Ha-Ras harvey rat sarcoma viral oncogene homolog (HRAS), epidermal growth factor receptor (EGFR), cysteinyl aspartate specific proteinase 3 (Caspase3), and prostaglandin-endoperoxide synthase 2 (PTGS2) as pivotal targets of butyroside anti-breast cancer (BC), suggesting that the PIK3CA-hsa-mir-139-5p-linc01278 and PIK3CA-hsa-mir-375 pathways may be closely related to the mechanism of syringin anti-BC. *In vitro* experiments confirmed that syringin inhibits BC cell proliferation and migration while promoting BC cell apoptosis through the above hub targets.

Angiogenesis plays a critical role in tumor growth and metastasis, making the inhibition of angiogenesis a promising approach for cancer therapy. Reverse molecular docking studies have identified syringin as a potential target for angiogenesis inhibition, which was further validated by a chorionic allantoic membrane assay. The study showed that the anti-angiogenic activity of syringin at concentrations of 100 μM and 200 µM was comparable to that of the positive control celecoxib at 200 µM (Aventurado et al., 2020). Furthermore, syringin has been found to inhibit angiogenesis regulatory enzymes such as transforming growth factor beta receptor 1 (TGF-βR1 kinase), human epidermal growth factor receptor 2 (HER2 kinase), EGFR kinase, fibroblast growth factor receptor 4 (FGFR4 kinase), and matrix metallopeptidase-2 (MMP-2) (Aventurado et al., 2023).

The chemical structure of syringin, a component of mustard alcohol, confers antioxidant activity, enabling it to combat free radical damage and exert antitumor effects. Specifically, carbonic anhydrase 9 (hCAIX) and carbonic anhydrase 2 (hCAXII) are crucial in acidifying the tumor environment and promoting tumor cell invasion and metastasis. Studies have demonstrated that syringin is a potent dual inhibitor of hCAIX and hCAXII, with the most promising outcomes associated with a phenol fraction (Costa et al., 2020). Furthermore, research indicates that syringin may potentially treat BC by increasing reactive oxygen species (ROS), inhibiting cancer cell growth, and significantly reducing tumor size (Lee et al., 2019).

### 6.3 Antihyperglycemic and antihyperlipidemic effects

**FIGURE 7:**
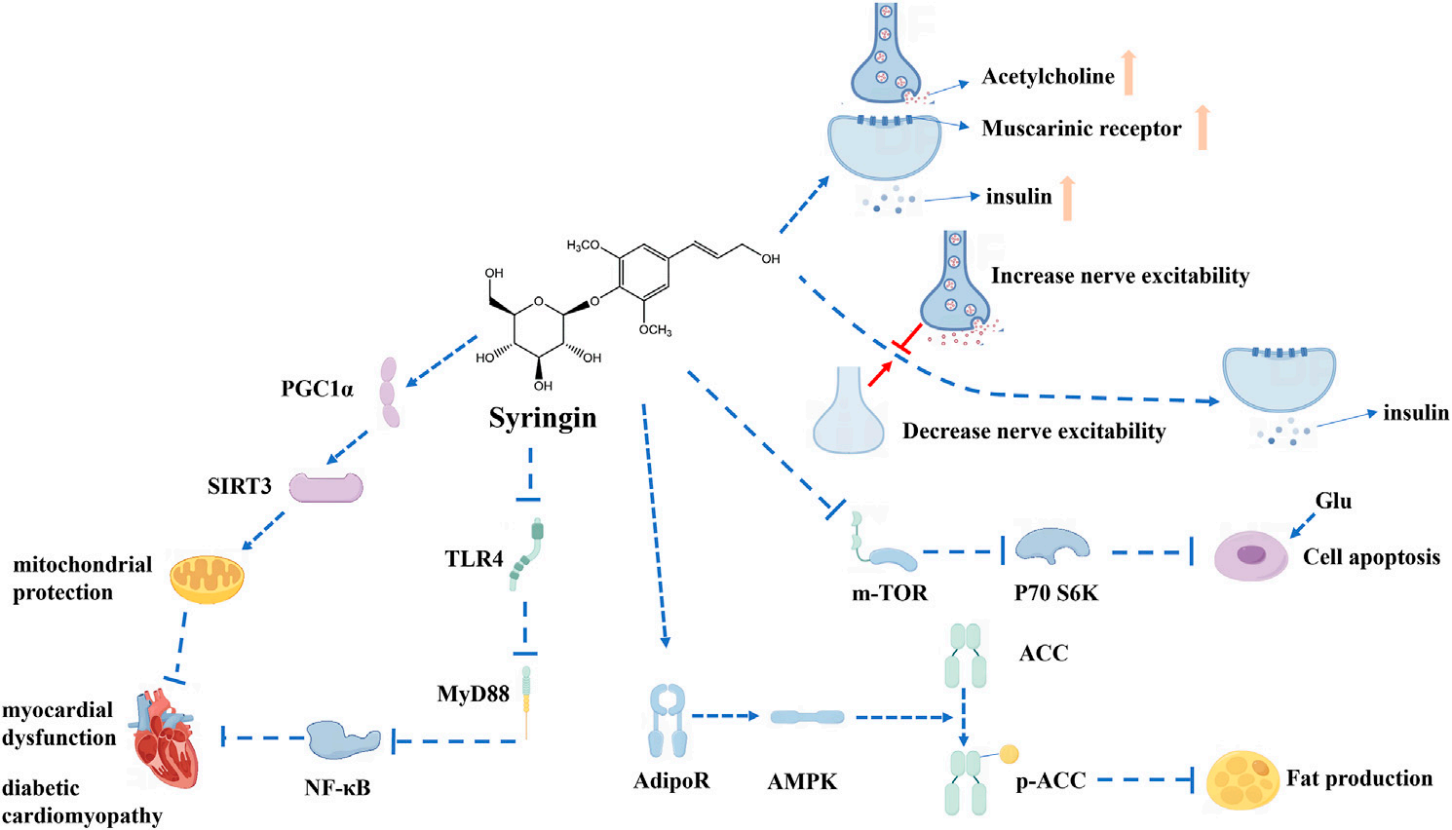
Mechanism of antihyperglycemic and antihyperlipidemic effects of syringin.

In recent years, the antihyperglycemic and antihyperlipidemic effects of syringin have garnered increasing attention from scholars. Syringin has shown particular promise in the treatment of type 1 diabetes mellitus (Niu et al., 2007; Niu et al., 2008b; Liu et al., 2008; Shen et al., 2020) and type 2 diabetes mellitus (Kim et al., 2017; Yao et al., 2021; Deng et al., 2023) as well as in correcting specific haematological parameters (Us et al., 2020) (Figure 7).

Research has indicated that syringin enhances the release of acetylcholine from nerve endings, stimulating muscarinic M3 receptors in pancreatic cells and increasing insulin release (Niu et al., 2007). It has also been suggested that the inhibition of activation of the mammalian target of rapamycin/ribosomal protein S6 kinase (mTOR/p70SK) pathway by syringin in high glucose states may play a role (Yao et al., 2023). Additionally, it has been observed that increased sympathetic nerve activity in awake animals may interfere with the insulin-modulating effects of syringin, suggesting that decreasing sympathetic tone could contribute to its efficacy (Niu et al., 2008a). Furthermore, combination therapy with syringin and tilianin has been found to treat diabetic cardiomyopathy through interactions with toll-like receptor 4/myeloid differentiation primary response gene 88/NF-κB/nucleotide-binding oligomerization domain-like receptor family, pyrin domain-containing 3 (TLR4/MyD88/NF-κB/NLRP3) and human peroxisome proliferator-activated receptor gamma coactivator one alpha/recombinant sirtuin 3/ROS (PGC1α/SIRT3/ROS) signaling pathways (Yao et al., 2021). However, it is insufficient to account for the mechanism of syringin and further study is required.

Studies have demonstrated that syringin is one of the most active lipocalin receptor two agonists (Sun et al., 2013), inhibiting inflammation, lipotoxicity, endoplasmic reticulum stress, and reducing insulin resistance (Kim et al., 2017). Moreover, it effectively promotes phosphorylation of Adenosine 5’-monophosphate (AMP)--activated protein kinase and acetyl coenzyme A carboxylase, inhibiting adipogenesis and promoting lipid metabolism. Therefore, syringin has the potential to be applied as an anti-obesity drug (Hossin et al., 2021).

### 6.4 Neuroprotection

Neurodegenerative diseases result from the loss of neurons and their myelin sheaths, which deteriorate over time, leading to dysfunction. These diseases can be categorized into acute neurodegenerative diseases (such as cerebral ischemia and brain injury) and chronic neurodegenerative diseases (including Alzheimer’s disease and Parkinson’s disease). Research has demonstrated that syringin possesses specific neuroprotective effects.

Syringin has been found to exert a protective effect against brain ischemia/reperfusion injury by reducing inflammation associated with cerebral ischemia. This protective mechanism is regulated through the Forkhead box O3 (FOXO3α)/NF-κB pathway. (Tan et al., 2021). In experimental rats, syringin decreased both inflammation reaction and cerebral damage in cases of cerebral ischemia/reperfusion injury. Furthermore, the neuroprotective properties of syringin may be linked to its inhibition of TLR4 (Liu et al., 2022). Additionally, syringin is considered an inhibitor of volume-regulated anion channel (VRAC). A study revealed that syringin moderately inhibited VRAC currents dose-dependently (Xu et al., 2023).

Bai et al. (2011) demonstrated that the ethyl acetate, n-butanol, and water fractions from the methanol extract of Siberian ginseng could protect against Aβ(25–35)-induced neuritic atrophy. Syringin was identified as one of the main active constituents responsible for this neuroprotective effect. It has been suggested that the mechanism involves reducing apoptosis, as evidenced by a decrease in caspase-3 activity and expression, reduction in cleaved poly ADP-ribose polymerase (PARP), and inhibition of deoxyribonucleic acid (DNA) fragmentation (Yang et al., 2010). Furthermore, syringin shows promise as a candidate agent for preventing and treating Parkinson’s disease by inducing autophagy through partially regulating the microRNA-34 Alpha/Sirtuin 1/Beclin-1 (miR-34α/SIRT1/BECN1) axis to prevent 6-oxidopamine hydrobromide (6-OHDA)-induced apoptosis and α-synuclein accumulation (Fu et al., 2023). Additionally, syringin has been found to prevent Aβ25–35 induced neurotoxicity via the microRNA-124-3p/B-cell lymphoma-2 (Bcl-2) homology three interacting domain death agonist (miR-124-3p/BID) pathway, suggesting its potential for broadening the pharmacological treatment options for Alzheimer’s disease (Zhang N. et al., 2021).

### 6.5 Hepatoprotection

The liver is crucial in maintaining physiological processes and is essential for life. It regulates blood volume, metabolizes nutrients, supports the immune system, maintains lipid and cholesterol balance, and breaks down exogenous compounds. Syringin is a potent antihepatotoxic drug that can restore enzyme activity in the microsomal enzyme system and inhibit lipid peroxidation. It promotes liver toxin metabolism and improves overall liver function.

Comparative metabolomics research has revealed that the antiviral effect of syringin in the hepatitis B (HBV) animal model is associated with arachidonic acid, citric acid, ornithine, L-lysine, L-glutamine, uric acid, pyruvic acid, and L-phenylalanine as potential therapeutic targets (Jiang et al., 2019). Studies have shown that syringin (25 and 50 mg/kg) has a protective and dose-dependent effect on LPS/D-GalN-induced liver injury in mice. This mechanism of action is related to its ability to increase antioxidant enzyme activity and inhibit inflammatory factor production (Hu et al., 2023). Additionally, syringin has been reported to provide potent protection against LPS/D-GalN-induced FHF by reducing lethality rates, alleviating hepatic pathological injury, inhibiting hepatocyte apoptosis, and reducing hepatic inflammatory responses. These protective effects are likely mediated by suppressing TNF-α production through NF-κB inhibition (Mao et al., 2023).

### 6.6 Anti-radiation

Radiation has the potential to cause extensive DNA damage in the human body, leading to severe harm to biological systems through cell death or the induction of mutations in surviving cells, which can ultimately result in cancer (Bugris et al., 2019). Research indicates that syringin exhibits a protective effect against radiation-induced damage.

Specifically, syringin has been shown to ameliorate cytokine regulation disorders induced by low-dose radiation 0.6 Gy (Gy) in mice and effectively reduce reproductive system damage, suggesting its potential as a protective agent against such damage caused by low-dose radiation exposure (Hu et al., 2023). Furthermore, syringin demonstrates a specific protective effect on spleen and thymus damage resulting from low-dose radiation exposure (1 Gy), potentially increasing Nrf2 levels via protein kinase B (AKT) and enhancing Nrf2-mediated antioxidant effects in mouse immune organs (Mei, 2023). In a study by Song et al. (Song et al., 2022), a brain injury model was generated using ^60^Co-γ ray radiation at 4 Gy in mice. The findings revealed that syringin significantly improved radiation-induced cognitive dysfunction and decreased monoamine oxidase levels to prevent brain damage.

### 6.7 Cardioprotection

The incidence of heart disease has significantly increased in recent years, leading to high morbidity, mortality, recurrence, disability, and economic burden. Numerous researchers have focused on identifying the molecular mechanism of cardiac injury to develop new drugs for preventing heart failure. Syringin shows potential in the treatment of myocardial ischemia, and further research into its mechanism of action in cardiovascular disease will provide more options for patient care.

Studies have demonstrated that long-term oral administration of syringin attenuates the development of cardiac hypertrophy induced by pressure overload and improves cardiac functions. The protective effects of syringin may be attributed to its inhibition of adenosine-activated protein kinase α (AMPKα) and autophagy-related signaling pathways (Li et al., 2017). In Langendorff-perfused rabbit hearts, the application of syringin (200 μM) or tetrodotoxin (2 μM) substantially decreased incidences of atrial fibrillation (AF), ventricular fibrillation, and heart death induced by anemone toxin II. These results suggest that syringin exerts anti-AF actions mainly via blocking late sodium current, providing a basis for new clinical applications in pharmacology (Zhang P. P. et al., 2021). *In vitro* studies have shown that syringin (10 μg/mL) can significantly stimulate intestinal bacteria to produce short-chain fatty acids, which may contribute to the effect on myocardial ischemia. The impact on isovaleric acid production was particularly significant, with a 27.9% increase at 24 h; propionic acid concentration also increased by 9.5%. Butyric acid showed a lesser extent increase at 16.3% at 24 h, with no significant difference observed (Yu et al., 2022).

### 6.8 Bone protection

The process of bone metabolism involves the breakdown of old or mature bone (catabolism) and the formation of new bone (anabolism), which is carried out by osteoclasts and osteoblasts, respectively. Any disruption in this process can lead to a loss of bone mass or osteoporosis. Syringin has been identified as a potent multi-targeted drug for bone formation against osteoporosis.

Research studies have demonstrated that syringin significantly increases alkaline phosphatase activity by 131.01% and enhances mineral deposition by 139.39%. It also regulates autophagy and the bone morphogenetic protein-2 (BMP-2) signaling pathways (Imtiyaz et al., 2020). Furthermore, syringin exhibits strong anti-osteoporosis effects in ovariectomized mice, with its underlying molecular mechanism possibly being the NF-κB and human phosphoinositide-3 kinase/protein kinase B (PI3K/AKT) signaling pathways (Liu et al., 2018). In addition, some scholars have found that syringin improves histopathological damage to articular cartilage in rats with anterior cruciate ligament transection-induced osteoarthritis by inhibiting inflammatory responses, degradation of the extracellular matrix, and activation of the proto-oncogene Wnt-1/recombinant beta-catenin (Wnt/β-catenin) signaling pathway (Qiao et al., 2022).

## 7 Drug safety

Toxicity studies on Chinese medicines and their main components provide a scientific basis for determining safety limits and taking preventive and control measures. In ancient books, herbs are classified into upper, middle, and lower grades. The upper grade is considered a life-extending, non-toxic medicine. *Acanthopanax senticosus* (Rupr. et Maxim.) Harms were first recorded in *Shennong’s Classic of the Materia Medica* and classified as an upper-grade herb (Lee et al., 2019).

In this study, computational tools were used to predict the toxicity of syringin. The blockage of the human Ether-a-go-go-related gene (hERG) K+ channels is closely associated with lethal cardiac arrhythmia. The pred-hERG predicted syringin as non-cardiotoxic with a 50% confidence value. *General Unrestricted Structure-Activity Relationships* predicted the median lethal dose values for rats with four types of administration (oral, intravenous, intraperitoneal, subcutaneous, and inhalation): 0.923, 0.889, 0.503, and 1.208 mmol/kg, respectively. According to the acute rodent toxicity classification of chemicals by the *Organisation for Economic Co-operation Project*, syringin was predicted to be non-toxic for intravenous and subcutaneous routes of administration and categorized in toxicity class 5 for intraperitoneal and oral routes of administration. Regarding environmental toxicity, the predicted bioaccumulation factor is less than five, which indicates low environmental toxicity according to *Annex D of the Stockholm Convention* (Ahmed et al., 2021). Additionally, syringin is not predicted to be an inhibitor of cytochrome P450 (CYP), making it less likely to cause adverse drug reactions. However, it was reported as a weak inhibitor of CYP2C9 and CYP2E1 in rat liver microsomes, and it remains to be investigated whether the weak inhibitory effect on these two CYP isoforms is clinically relevant (Guo et al., 2014).

## 8 Preparations and applications

**FIGURE 8:**
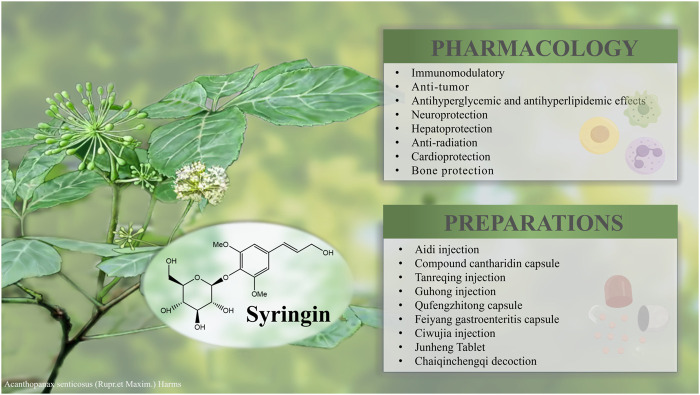
Pharmacological effects and clinical applications of syringin.

Syringin is a key component in numerous pharmaceuticals, nutraceuticals, and food products. It plays a crucial role in the treatment of cancer, cardiovascular and cerebrovascular diseases, inflammation, as well as liver and kidney deficiencies (Table 5) (Figure 8). However, there is currently limited analysis of the active ingredients in these formulations, making it difficult to determine the specific effects of syringin.

**TABLE 5:** Traditional Chinese medicine preparations with syringin.

Product name	Ingredient	Indication	Execution standards	Ref.
Aidi injection	*Mylabris phalerata* Pallas, *Panax ginseng* C. A. Mey., *Astragalus membranaceus* (Fisch.) Bge. var. mongholicus (Bge.)Hsiao, *A. senticosus* (Rupr.et Maxim.) Harms, et al	Lung cancer, liver cancer, and colorectal cancer	WS3-B-3809-99-2002	Yang et al. (2022)
Compound cantharidin capsule	*M. phalerata* Pallas, *P. ginseng* C. A. Mey., *A. membranaceus* (Fisch.) Bge. var. mongholicus (Bge.) Hsiao, *A. senticosus* (Rupr. et Maxim.) Harms, et al	Rectal cancer, lung cancer, malignant lymphoma, primary liver cancer, gynecological malignant tumors	WS3-B-3272-98	Sun et al. (2017)
Tanreqing injection	*A. membranaceus* (Fisch.) Bge. var. mongholicus (Bge.) Hsiao, *Saiga tatarica* Linnaeus, *Lonicera japonica* Thunb., *Forsythia suspensa* (Thunb.) Vahl, et al	Respiratory system diseases	YBZ00912003-2007Z-2009-2012	Wang et al. (2021b)
Guhong injection	Aceglutamide, *Carthamus tinctorius* L	Cerebrovascular disease	WS-10001(HD-1506)-2004-2012	Wang et al. (2022b)
Qufengzhitong capsule	*Clematis chinensis* Osbeck, *Taxillus chinensis* (DC.) Danser, *Erodium stephanianum* Willd., *Angelica pubescens* Maxim.f. biserrata Shan et Yuan, *C. tinctorius* L	Neurogenic pain, aching lumbus, and knees	WS3-0049 (Z-049)-2001(Z)	Liao et al. (2022)
Feiyang gastroenteritis capsule	*Euphorbia hirta* L., *Polygonum chinense* L., *I. rotunda* Thunb	Bacterial dysentery and gastroenteritis	YBZ09622008	Li et al. (2019)
Ciwujia injection	*A. senticosus* (Rupr. et Maxim.) Harms	Liver and kidney deficiency, cardiovascular and cerebrovascular diseases	WS3-B-3425-98	Fan et al. (2014)
Junheng Tablet	*A. senticosus* (Rupr. et Maxim.) Harms, *Cistanche deserticola* Y. C. Ma, *P. quinquefolium* L., *Salvia miltiorrhiza* Bge	Anti-fatigue	-	Wang et al. (2020)
Chaiqinchengqi decoction	*L. japonica* Thunb. Fl. Jap., *Taraxacum mongolicum* Hand. -Mazz., *Bupleurum chinense* DC., *A. membranaceus* (Fisch.) Bge. var. mongholicus (Bge.) Hsiao, et al	Acute edematous pancreatitis	-	Liang et al. (2021)

Aidi injection is among the Chinese medicinal injections approved by the *China Food and Drug Administration*. It has been assessed for its adjuvant beneficial effects on tumor survival, tumor response, quality of life, and reduction of radiotherapy side effects. This evaluation has focused on lung, liver, and colorectal cancers. Aidi injection has been recognized as one of the most competitive products in cancer therapy in China (Yang et al., 2022). The compound cantharidin capsule is primarily used for treating rectal cancer, lung cancer, malignant lymphoma, primary liver cancer, and gynecological malignancies (Sun et al., 2017). These two formulations utilize *M. phalerata* Pallas as the primary therapeutic agent to exert antidotal effects and dispel pathogenic factors. *Acanthopanax senticosus* (Rupr.et Maxim.) Harms addresses deficiencies while calming the spirit, fortifying the kidneys, and reinforcing essence. Syringin may modulate immunity, combat tumors, protect neurons, and safeguard liver and kidney function (Dai et al., 2024).

Guhong injection is a novel combination of Chinese and Western medicine, approved by the *State Food and Drug Administration of China* in 2003. It has been widely used in China to treat cerebrovascular diseases, especially ischemic stroke. Syringin is considered as one of the chemical compositions (Wang Y. et al., 2022). While syringin has demonstrated cardiovascular and neuroprotective effects, current research does not establish it as the primary active ingredient. Ciwujia injection is a brownish-red sterilized solution of *A. senticosus* (Rupr. et Maxim.) Harms extracted by the method of “hydrotropic alcohol precipitation”. It is clinically used for transient ischemic attacks, cerebral arteriosclerosis, cerebral thrombosis, and cerebral embolism caused by liver and kidney insufficiency. It is also used for coronary heart disease, angina pectoris combined with neurasthenia, and menopausal syndrome (Fan et al., 2014). Syringin may be involved in hepatorenal protection, cardiovascular and cerebrovascular health, and immune regulation. However, there is still a lack of relevant studies to confirm these potential effects.

Tanreqing injection is widely utilized in clinical practice to manage respiratory disorders. Research has indicated that syringin is among the active compounds (Wang L. et al., 2021). Feiyang gastroenteritis capsule, a proprietary Chinese medicine, is extensively employed to treat bacterial dysentery and acute and chronic gastroenteritis. Studies have demonstrated that one of its primary components, syringin, possesses anti-inflammatory properties (Li et al., 2019). Chaiqinchengqi decoction has been an efficacious therapy for acute pancreatitis at West China Hospital for many years. The protective impact of syringin on pancreatic acinar cell death was found to be positively associated (Liang et al., 2021).

Junheng Tablet is a health food with syringin as its signature ingredient. According to Wang et al. (2020), syringin can effectively accelerate the removal of metabolic wastes from the body and maintain normal physiological function, thus delaying the onset of fatigue and promoting faster recovery of physical fitness.

The Qufengzhitong capsule is clinically utilized to treat neuropathic, lumbar, and knee pain. Syringin is identified as one of its seven active ingredients (Liao et al., 2022).

## 9 Pharmacokinetics

Syringin is rapidly eliminated *in vivo* and exhibits low bioavailability. In rats, the area under the curve (AUC_0-T_) was (3.26 ± 0.60) μg/h/mL, with a plasma half-life time (t_1/2_) of (0.41 ± 0.04) h and elimination rate constant (Ke) of (1.85 ± 0.16) h^− 1^ (Li et al., 2007). Tan and Jia (2008) conducted *in vivo* intestinal perfusion experiments on rats and observed variations in drug absorption and corrected intestinal wall permeability of syringin across different intestinal segments, with the following order: duodenum > jejunum > ileum > colon. It was noted that syringin underwent degradation by intestinal enzymes, with the levels of degradation as follows: duodenum > jejunum > ileum > colon. Additionally, syringin was not detected in plasma and bile, suggesting potential metabolism by intestinal enzymes within the intestine, leading to low bioavailability. Yang et al. (2022) discovered that the rat intestinal flora plays a significant role in metabolizing syringin. They analyzed the incubation solution of rat intestinal flora isolated from 0 to 48 h with syringin, revealing that 81% of the syringin was metabolized within 12 h, with erucic alcohol identified as the primary metabolite. By 24 h, syringin was completely metabolized, and the primary metabolite was dextroseyl resinoid phenol; however, erucic alcohol could not be detected after 24 h. Fan et al. (2014) preliminary explored the co-existing component of p-syringin in Ciwujia injection and its pharmacokinetic effect. Male rats were intravenously administered with a syringin monomer and a corresponding dose of Ciwujia injection. The plasma syringin concentration was determined at different time points using liquid chromatography-tandem mass spectrometry (LC-MS/MS). The AUC_0-∞_ values of syringin were (429.5 ± 25.6) and (721.0 ± 81.8) µg/h/L, respectively, and the plasma clearance (CL) of syringin was (3.3 ± 0.2) and (2.0 ± 0.2) L/h/kg, respectively, both showing significant differences. It was observed that the coexisting components in Ciwujia injection can increase the accumulation of syringin in rats; however, further confirmation is required to elucidate the specific mechanism.

**FIGURE 9:**
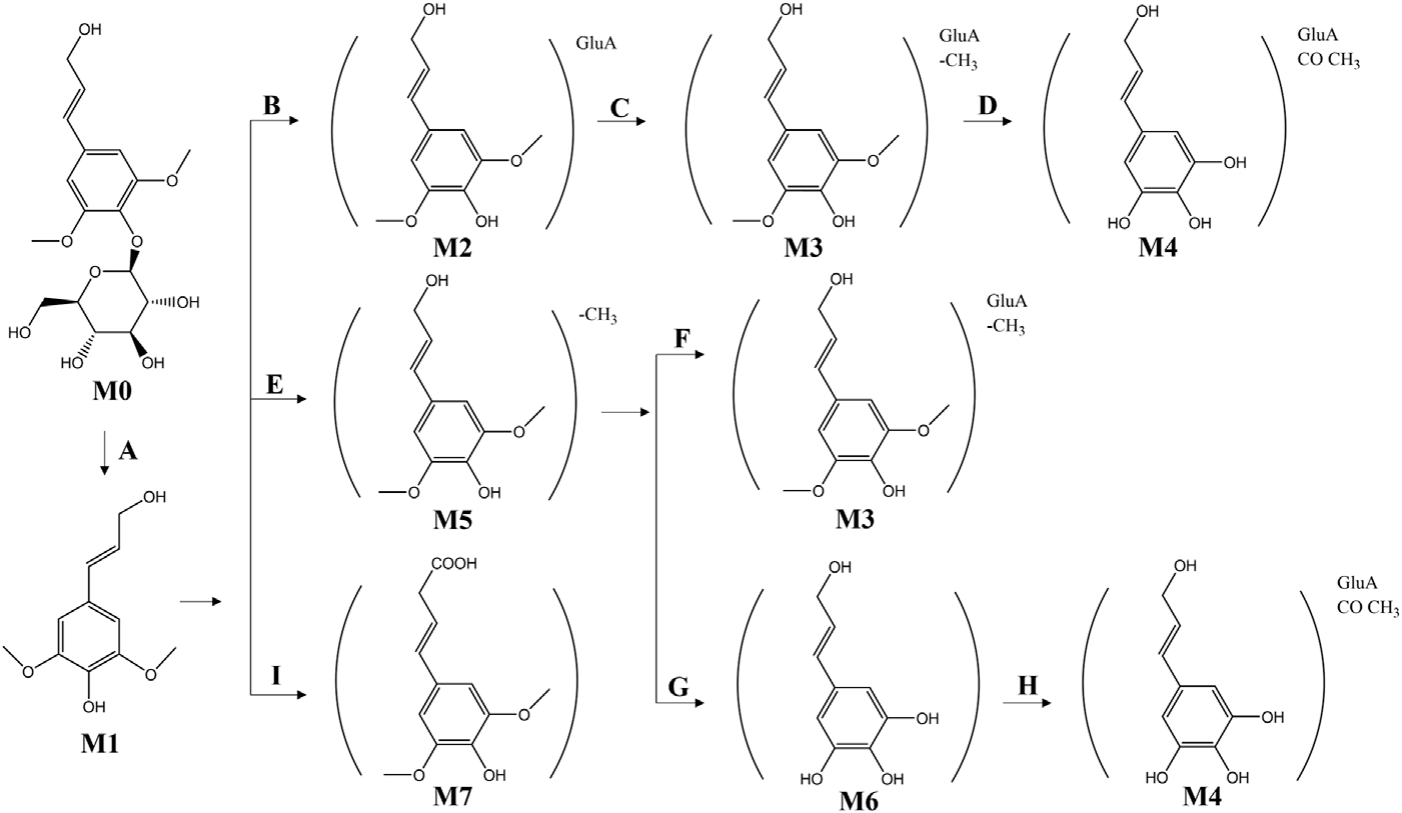
Potential metabolic biotransformation pathways and metabolites of syringin in rats. **(A)** (Deglycosylation); **(B)** (Glucuronidation); **(C)** (Demethylation); **(D)** (Demethylation + acetylation); **(E)** (Demethylation); **(F)** (Glucuronidation); **(G)** (Demethylation); **(H)** (Glucuronidation + acetylation); **(I)** (Hydroxylation + desaturation).

Lu et al. (2012) analyzed rat plasma using HPLC/quadrupole time-of-flight mass spectrometry (QTF-MS)/MS and automated data analysis. A total of 11 metabolites were detected, with no syringin being found. In rats, syringin (M0) was deglycosylated (Process A) to obtain the glycoside erucinol (M1). Subsequently, M1 was further glucuronidated (Process B) to obtain the glucuronidated erucinol (M2), which was then demethylated (Process C) to produce the demethylated glucuronidated erucinol (M3). The product M4 was obtained from M3 through further demethylation and acetylation (Process D). Additionally, after deglycosylation of M0, it can be further hydroxylated and desaturated to obtain the product M7 through Process I. Simultaneously, demethylation and glucuronidation of M0 led to the production of demethylated glucuronide erucicol M3 through Processes E and F; this could then undergo further glucuronidation and acetylation via Processes G and H to yield a new product. The *in vivo* metabolic pathway of syringin is illustrated in Figure 9. The low bioavailability of syringin may be closely related to its metabolic processes in organisms. Further consideration and research are warranted to explore the transformation of its relationship with mustelianol and dextrose resinoid phenol and whether its metabolism mode affects its efficacy (Wang F. et al., 2021).

## 10 Discussion

Based on the rich pharmacological effects of syringin, its further development and application require efforts from multiple aspects.

### 10.1 Need for standardization in pharmacological research

Many polyphenols including phenylpropanoids like syringin have been reported may interfere with formazan formation critical to the MTT or MTS methods, resulting in inaccurate positive outcomes (Wang P. et al., 2010). CCK-8 and other methods have been used to test the pharmacological activities of *in vitro* experiments.

In silico analysis has shown potential in predicting the pharmacological effects and mechanisms of syringin. However, the databases upon which network pharmacology relies require improvement in terms of accuracy, adaptability, and reliability. It is important to note that while this method is not fabricated, its evidence level is lower compared to experimental and clinical research (Fatima et al., 2022).

Since a long time ago, the activities of syringin have been tested in a lot of experiments relating to mice, rats, and rabbits. Unfortunately, there were some design defects in the early pharmacological studies, including the absence of positive control drugs and excessively high dose settings. To better promote the development of syringin, standardized pharmacological studies are necessary.

### 10.2 The mechanism of syringin relating to immune regulation and anti-tumor effect needs to be exposed

Over the past decade, immunotherapy, such as programmed death-1 (PD-1), supplied a breakthrough in cancer treatment (Chow et al., 2022). *Fuzheng* and *quxie* is a fundamental approach in traditional Chinese medicine for tumor therapy. It focuses on enhancing the body’s immunity in contrast to the tumor. More and more research has shown that many tonic Chinese medicines have anti-tumor effects by boosting immune cell activity and exhibiting collaborative and enhancement effects with PD-1 (Chen F. Q. et al., 2021). As to syringin, this kind of experiment was rare and highly recommended for further study.

### 10.3 Clinical data should be collected in standardized experiments

Compared to cell and animal experiments, the clinical trial is more convincing to confirm the effectiveness and safety of a new drug. Despite the Aidi injection which contains syringin as the main component, has annual sales of more than one billion in China (Ding, 2024), it still lacks a large population, and multi-species randomized, double-blind, controlled clinical trials. Therefore, further improvement of clinical data is crucial to promote its application in cancer patients worldwide.

## 11 Conclusion

Since Tu Youyou was awarded the “2015 Nobel Prize in Physiology or Medicine”, there has been significant progress in developing natural products extracted from Chinese herbs, which have contributed to treating diseases. Syringin exhibits rich pharmacological effects and high safety, making its further development and utilization of great significance for medicine and clinical applications. Therefore, stronger support is needed for its further development and utilization to promote economic growth while safeguarding public health. This paper summarizes relevant research reports on syringin as a reference for addressing the issues above.
